# Ethnic Disparities in Hepatitis B Virus Infection Among 1 Million Reproductive-Age Couples Preparing for Pregnancy in the Rural Yunnan, China: A Population-Based Cross-Sectional Study

**DOI:** 10.3389/fmed.2021.799873

**Published:** 2022-01-26

**Authors:** Wenzhan Jing, Yanling Yuan, Min Liu, Hanfeng Ye, Cai Kong, Jue Liu, Yu Wu

**Affiliations:** ^1^Department of Epidemiology and Biostatistics, School of Public Health, Peking University, Beijing, China; ^2^Reproductive Epidemiology Laboratory, Yunnan Population and Family Planning Research Institute, Kunming, China

**Keywords:** hepatitis B virus, ethnic, disparity, reproductive-age, China

## Abstract

**Introduction:**

Hepatitis B is a potentially life-threatening liver infection caused by hepatitis B virus (HBV) and China has the largest disease burden. We aim to understand the ethnic disparities in HBV infection among the married reproductive-age couples planning for pregnancy in Yunnan, a multiethnic province in Southwest China, to increase the health equities within the hepatitis response in China.

**Methods:**

A population-based cross-sectional study was performed. Couples aged 20–49 years in rural Yunnan were enrolled through the National Free Preconception Health Examination Project from Jan 2014 to Dec 2019. HBsAg-positive couples were defined as couples in which one or both were HBsAg-positive, and HBsAg- and HBeAg-positive couples were defined as couples in which one or both were HBsAg- and HBeAg-positive. The HBV prevalence of positive couples was estimated by ethnicity. Multivariate logistic regression analyses were used to assess the association between ethnicity and HBsAg status.

**Results:**

Overall, 63,513 of 1,060,643 couples (5.99%, 95% CI, 5.94%−6.03%) were HBsAg-positive, and 15,898 of 63,513 HBsAg-positive couples (25.03%, 95% CI 24.69%−25.37%) were HBsAg- and HBeAg-positive couples in rural Yunnan. The highest prevalence of HBsAg-positive couples was in the Miao and Miao ethnicity (12.04%) and Zhuang and Zhuang ethnicity (9.76%), and the risk of HBV infection of wives/husbands in these ethnic groups was significantly higher than that in the Han and Han ethnicity. Additionally, the HBsAg prevalence in wives/husbands has increased with the positive status of HBsAg and HBeAg of their spouses.

**Conclusion:**

The HBV prevalence in reproductive-age couples was intermediate (6% of 1 million couples) in rural Yunnan, China, with the highest in the Miao and Zhuang ethnicities. There are still large ethnic disparities in HBV infection in China. Therefore, China should make great efforts, especially giving priority to ethnic minorities and taking positive couples as an important unit of care, to equitably eliminate the HBV intrafamilial transmission.

## Introduction

Hepatitis B is a potentially life-threatening liver infection caused by the hepatitis B virus (HBV) (1). It accounts for a major global public health threat and high mortality from cirrhosis and liver cancer (1). The WHO estimated that 296 million people were living with chronic HBV infection, resulting in an estimated 820,000 deaths worldwide in 2019 (2). China has the largest disease burden of hepatitis B (3), with an estimated 70 million people living with HBV infection and 162,000 deaths related to hepatitis B in 2019 (4, 5). Therefore, China will be a major contributor to achieving the WHO goals of eliminating hepatitis B worldwide by 2030 (3).

Hepatitis B virus is most commonly transmitted from mother to child during birth and delivery (1). China has made large progress in the hepatitis response in preventing the HBV mother-to-child transmission due to the high coverage of timely birth-dose and three-dose of hepatitis B vaccines in recent decades (4, 6). HBV is also transmitted by contact with contaminated blood or other body fluids, such as saliva and menstrual, vaginal, and seminal fluids (1). Therefore, reproductive-age couples planning for pregnancy are the key stakeholders in preventing HBV intrafamilial transmission (7), because using barrier protective measures (condoms) to protect against transmission is infeasible. If one of the couples is infected with HBV, the source of HBV transmission exists in the household settings, not only increasing the risk of HBV transmission to the other spouse but also increasing the risk of mother-to-child transmission (7). Additionally, the number of couples preparing for pregnancy will increase in China, because the government recently announced the three-child policy, allowing all couples to have up to three children (8). Therefore, studies focusing on the HBV prevalence among married couples as a unit are necessary for China.

Some priority populations, either most severely affected or at higher risk of HBV infection, do not garner extra attention in China, so there is still a long way to go to comprehensively eliminate hepatitis B. For example, there are 56 ethnic groups that are officially recognized in China, with Han ethnicity as the dominant and 55 other ethnic groups as ethnic minorities (9). Previous studies reported that the HBV prevalence in some minorities was higher than that in Han ethnicity (10–12). However, there are no published studies focusing on ethnic disparities in HBV infection among couples planning for pregnancy, which may help us to assess the priority ethnic groups for HBV intrafamilial prevention and control and to formulate evidence-based policies and data for action, thereby increasing health equities within the hepatitis response in China.

Yunnan, a multiethnic and relatively poor province in Southwest China (13), can be regarded as a unique province to explore the intrafamilial interventions with ethnic characteristics. In this study, we aimed to conduct a population-based cross-sectional study to investigate the ethnic disparities in HBV prevalence among reproductive-age couples preparing for pregnancy in rural Yunnan, China, to provide reliable data for better development of rational strategies targeting intrafamilial HBV transmission for equitably eliminating hepatitis B.

## Methods

### Study Design and Participants

A population-based and cross-sectional study was performed among married couples of reproductive ages preparing for pregnancy by the National Free Preconception Health Examination Project (NFPHEP) from Jan 1, 2014, to Dec 31, 2019, in rural Yunnan, China. Detailed information on the NFPHEP was previously described elsewhere (14). Briefly, NFPHEP was launched by the Chinese government to provide free preconception health examinations and counseling services for married couples in rural areas who planned to become pregnant (14). After obtaining a written informed consent form from participants, a standardized family heath file was completed by locally trained health workers using the questionnaire survey and medical examination (14).

In the present analysis, the inclusion criteria were as follows: (1) couples were enrolled by the NFPHEP from Jan 1, 2014, to Dec 31, 2019; (2) couples were living in rural Yunnan, China; (3) couples were aged 20–49 years; and (4) couples had preconception health examinations. The exclusion criteria were as follows: (1) one or both of the couples had missing demographic information; (2) one or both of the couples did not test for HBV markers; and (3) one or both of the couples' records of hepatitis B surface antigen (HBsAg) or hepatitis B e antigen (HBeAg) status were incomplete.

### Questionnaire

A standardized questionnaire was used to collect basic information of the couples, including ethnicity, age, education level, occupation, and residence address. Han, the dominant ethnicity, made up ~66.88% of the total population in Yunnan (13). According to the ethnicity of husbands and wives, the included couples were grouped into Han and Han, Han and Minority, Minority and Minority (including Yi and Yi, Dai and Dai, Miao and Miao, Hani and Hani, Zhuang and Zhuang, Bai and Bai, Lisu and Lisu, Hui and Hui, and others). The age of the couple was calculated as the mean age of the husband and wife and grouped into 20–29, 30–39, and 40–49 years old. The education level of the couple was represented by the highest education level of the husband and wife, and divided into primary school or below, junior high school, senior high school or equivalent, and college or equivalent/higher. The occupation of the couple was classified into both farmers, one farmer, and others. The socioeconomic status (SES) of the couple was measured by residential addresses and separated into living in impoverished or non-impoverished counties.

For SES, 88 of 129 counties in Yunnan were identified as impoverished counties by the Chinese government in 2014 (15), based on indicators that are highly related to the degree of impoverishment, such as the per capita county GDP and per capita rural net income, and consideration of minority ethnic area and border areas (16). The exclusion criteria for impoverished counties were as follows: the impoverished incidence was lower than 3%; the disposable income per capita of the rural residents reached 70% of that in the province; 97% of villages had asphalt (cement) roads; the penetration rate of rural tap water was over 90%; all of the households accessed electricity; 97% of the households had safe housing; more than 97% of the poor people participated in the new rural cooperative medical care and critical illness insurance (17). In this study, if the counties officially withdrew from the impoverished counties (18), they were divided into the non-impoverished counties in the next year.

### Serologic Testing

Venous blood of the couples was obtained and was immediately sent to local laboratories to separate and store at −30°C (14). All serum specimens were tested with ELISA kits for HBV markers: HBsAg, an antibody against HBsAg (anti-HBs), hepatitis B e antigen (HBeAg), the antibody against HBeAg (anti-HBe), and the antibody against HBcAg (anti-HBc) (14). These local laboratories were affiliated with medical institutions under qualified quality control mechanisms and made their own choices on reagent kits (14). All reagent kits selected were tested by the National Center of Clinical Laboratories for Quality Inspection and Detection with reference reagents produced by Abbott (Abbott Park, IL, USA) (14). The sensitivity, specificity, and κ value of the selected reagents from all counties were higher than 95% (14). The National Center of Clinical Laboratories for Quality Inspection and Detection also performed an external quality assessment two times a year to evaluate and ensure the consistency of the results (14).

HBsAg positivity indicated that participants carried HBV and were thus infectious. In the present study, the HBsAg status of the couple was divided into only husband positive, only wife positive, and both positive, and defined as follows: the prevalence of only husband HBsAg positive = $\frac{couples\;only\;husband\;HBsAg\;positive}{all\;couples}\times 100\%$, the prevalence of only wife HBsAg positive = $\frac{couples\;only\;wife\;HBsAg\;positive}{all\;couples}\times 100\%$, the prevalence of both HBsAg positive = $\frac{couples\;both\;husband\;and\;wife\;HBsAg\;positive}{all\;couples}\times 100\%$. Additionally, HBsAg-positive couples were defined as couples in which one or both of them were positive for HBsAg, and the prevalence of HBsAg-positive couples was calculated as $\frac{couples\;one\;or\;both\;of\;them\;HBsAg\;positive}{all\;couples}\times 100\%$.

HBeAg positivity in HBsAg-positive participants indicated a high level of infectiousness. In the present study, HBsAg- and HBeAg-positive couples were defined as couples in which one or both were HBsAg- and HBeAg-positive. The proportion of HBsAg- and HBeAg-positive couples among couples only husband HBsAg-positive, only wife HBsAg-positive, and both HBsAg-positive was defined as: $\frac{couples\;only\;husband\;HBsAg\;and\;HBeAg\;positive}{couples\;only\;husband\;HBsAg\;positive}\times 100\%$, $\frac{couples\;only\;wife\;HBsAg\;and\;HBeAg\;positive}{couples\;only\;wife\;HBsAg\;positive}\times 100\%$, and $\frac{couples\;one\;or\;both\;of\;them\;HBsAg\;and\;HBeAg\;positive}{couples\;both\;husband\;and\;wife\;HBsAg\;positive}\times 100\%$ respectively. Additionally, we defined the proportion of HBsAg- and HBeAg-positive couples among HBsAg-positive couples as $\frac{couples\;one\;or\;both\;of\;them\;HBsAg\;and\;HBeAg\;positive}{couples\;one\;or\;both\;of\;them\;HBsAg\;positive}\times 100\%$.

### Statistical Analysis

Proportions were used to describe the sociodemographic characteristics of the included couples. The prevalence of HBsAg and its 95% CIs were calculated by the entire study group and different ethnicities. The HBeAg proportion in HBsAg-positive couples and its 95% CIs were also calculated by the entire study group and different ethnicities. Crude odds ratios (cORs) and adjusted odds ratios (aORs) with 95% CIs were calculated by univariate and multivariate logistic regression analyses, respectively, to assess the association between the HBV status of spouses and HBsAg status of wives/husbands. The independent variable was the HBV status of spouses, including HBsAg-negative (as the reference), HBsAg-positive and HBeAg-negative, and HBsAg-positive and HBeAg-positive. The binary dependent variable was the HBsAg status of wives/husbands, with HBsAg-negative as the reference. Multivariate logistic regression was calculated using the enter method and adjusted by ethnicity, age group, education, occupation, SES, and check-in year of the couples. Additionally, multivariate logistic regression was also used to assess the association between ethnicity (Han and Han ethnicity as the reference) and HBsAg status of wives/husbands (HBsAg-negative as the reference), adjusted by age group, education, occupation, SES, and check-in year of the couples, as well as the HBV status of husbands/wives. Subgroup analyses were also performed by the age group of the couples (20–29, 30–39, and 40–49-year-old), adjusted by education, occupation, SES, and check-in year of the couples, as well as the HBV status of husbands/wives.

All statistical analyses were performed using Stata version 15.0. All *p*-values were two-sided, and the level of statistical significance was defined as *p* lower than 0.05.

### Patient and Public Involvement

Patients or the public were not involved in the design, or conduct, or reporting, or dissemination plans of our research.

## Results

### Sociodemographic Characteristics of the Included Couples

In this study, a total of 1,105,379 couples were included from 129 counties in rural Yunnan by the NFPHEP. Sociodemographic information from 1,075,369 of the 1,105,379 couples (97.29%) was complete. Serum samples from 1,065,916 couples were tested for HBV markers, and the HBsAg and HBeAg status from 1,060,643 couples was complete; thus, 95.95% of the couples (1,060,643/1,105,379) were included in our final analysis.

Approximately half of the included couples (49.65%) were of Han and Han ethnicity, 18.34% of them were of Han and Minority ethnicity, and 32.01% of them were of Minority and Minority ethnicity (Table 1). The mean age of 67.51% of couples was below 30 years old, 77.65% of couples had an education level of junior high school or lower, and 88.20% of couples were both farmers. For SES, 65.11% of the couples were living in impoverished counties in this study.

**Table 1:** Sociodemographic characteristics of the included reproductive-age couples.

**Characteristics**	**Couples (No.)**	**Proportion (%)**
Total	1,060,643	100.00
**Ethnicity**		
Han and Han	526,605	49.65
Han and Minority	194,488	18.34
Minority and Minority		
Yi and Yi	115,084	10.85
Dai and Dai	67,377	6.35
Miao and Miao	21,346	2.01
Hani and Hani	19,460	1.83
Zhuang and Zhuang	15,015	1.42
Bai and Bai	13,869	1.31
Lisu and Lisu	12,355	1.16
Hui and Hui	8,776	0.83
Others	66,268	6.25
**Age group (years**)		
20–29	716,002	67.51
30–39	303,408	28.61
40–49	41,233	3.89
**Education**		
Primary school or below	191,415	18.05
Junior high school	632,136	59.60
Senior high school or equivalent	133,499	12.59
College or equivalent/higher	103,593	9.77
**Occupation**		
Both farmers	935,440	88.20
Only one farmer	52,235	4.92
Both others	72,968	6.88
**Socioeconomic status**		
Impoverished county	690,590	65.11
Non-impoverished county	370,053	34.89
**Check year**		
2014	167,532	15.80
2015	179,478	16.92
2016	188,639	17.79
2017	193,537	18.25
2018	187,404	17.67
2019	144,053	13.58

### Total HBsAg/HBeAg Status in Reproductive-Age Couples

In this study, 34,747 couples (3.28%, 95% CI, 3.24%−3.31%) were only husband HBsAg positive, 26,044 couples (2.46%, 95% CI, 2.43%−2.49%) were only wife HBsAg positive, and 2,722 couples (0.26%, 95% CI, 0.25%−0.27%) were both HBsAg positive (Table 2). In total, 63,513 of 1,060,643 couples (5.99%, 95% CI, 5.94%−6.03%) were HBsAg-positive couples, one or both of whom tested positive for HBsAg.

**Table 2:** Ethnic disparities in hepatitis B surface antigen (HBsAg) and hepatitis B e antigen (HBeAg) status in reproductive-age couples.

**Ethnicity**	**Total No**.	**Only Husband**				**Only Wife**				**Husband and wife**				**Positive couple**			
		**HBsAg+**		**HBsAg+ and HBeAg+** ^**1**^		**HBsAg+**		**HBsAg+ and HBeAg+** ^**2**^		**HBsAg+**		**HBsAg+ and HBeAg+** ^**3**^		**HBsAg+**		**HBsAg+ and HBeAg+** ^**4**^	
		**No**.	**% (95% CI)**	**No**.	**% (95% CI)**	**No**.	**% (95% CI)**	**No**.	**% (95% CI)**	**No**.	**% (95% CI)**	**No**.	**% (95% CI)**	**No**.	**% (95% CI)**	**No**.	**% (95% CI)**
Han and Han	526,605	15,042	2.86 (2.81–2.90)	3,331	22.14 (21.48–22.82)	11,575	2.20 (2.16–2.24)	2,800	24.19 (23.41–24.98)	1,069	0.20 (0.19–0.22)	437	40.88 (37.91–43.89)	27,686	5.26 (5.20–5.32)	6,568	23.72 (23.22–24.23)
Han and Minority	194,488	6,560	3.37 (3.29–3.45)	1,416	21.59 (20.59–22.60)	4,876	2.51 (2.44–2.58)	1,235	25.33 (24.11–26.57)	476	0.24 (0.22–0.27)	194	40.76 (36.31–45.32)	11,912	6.12 (6.02–6.23)	2,845	23.88 (23.12–24.66)
Minority and Minority																	
Miao and Miao	21,346	1,312	6.15 (5.83–6.48)	406	30.95 (28.45–33.53)	1,085	5.08 (4.79–5.39)	407	37.51 (34.62–40.47)	173	0.81 (0.69–0.94)	97	56.07 (48.33–63.59)	2,570	12.04 (11.61–12.48)	910	35.41 (33.56–37.29)
Zhuang and Zhuang	15,015	766	5.10 (4.76–5.47)	263	34.33 (30.97–37.82)	601	4.00 (3.69–4.33)	212	35.27 (31.45–39.24)	98	0.65 (0.53–0.79)	51	52.04 (41.71–62.24)	1,465	9.76 (9.29–10.24)	526	35.90 (33.44–38.42)
Hani and Hani	19,460	858	4.41 (4.12–4.71)	157	18.30 (15.77–21.05)	580	2.98 (2.75–3.23)	160	27.59 (23.98–31.42)	108	0.55 (0.46–0.67)	42	38.89 (29.66–48.75)	1,546	7.94 (7.57–8.33)	359	23.22 (21.14–25.41)
Dai and Dai	67,377	2,753	4.09 (3.94–4.24)	676	24.56 (22.96–26.21)	1,763	2.62 (2.50–2.74)	466	26.43 (24.39–28.56)	231	0.34 (0.30–0.39)	116	50.22 (43.59–56.84)	4,747	7.05 (6.85–7.24)	1,258	26.50 (25.25–27.78)
Lisu and Lisu	12,355	414	3.35 (3.04–3.68)	90	21.74 (17.86–26.03)	324	2.62 (2.35–2.92)	106	32.72 (27.63–38.12)	27	0.22 (0.14–0.32)	10	37.04 (19.40–57.63)	765	6.19 (5.77–6.63)	206	26.93 (23.81–30.22)
Yi and Yi	115,084	3,536	3.07 (2.97–3.17)	855	24.18 (22.78–25.63)	2,714	2.36 (2.27–2.45)	759	27.97 (26.28–29.70)	221	0.19 (0.17–0.22)	94	42.53 (35.93–49.34)	6,471	5.62 (5.49–5.76)	1,708	26.39 (25.32–27.49)
Hui and Hui	8,776	210	2.39 (2.08–2.73)	56	26.67 (20.82–33.19)	144	1.64 (1.39–1.93)	34	23.61 (16.94–31.40)	21	0.24 (0.15–0.37)	7	33.33 (14.59–56.97)	375	4.27 (3.86–4.72)	97	25.87 (21.51–30.61)
Bai and Bai	13,869	317	2.29 (2.04–2.55)	50	15.77 (11.94–20.26)	186	1.34 (1.16–1.55)	50	26.88 (20.66–33.86)	12	0.09 (0.04–0.15)	8	66.67 (34.89–90.08)	515	3.71 (3.40–4.04)	108	20.97 (17.53–24.75)
Others	66,268	2,979	4.50 (4.34–4.66)	657	22.05 (20.58–23.59)	2,196	3.31 (3.18–3.45)	541	24.64 (22.85–26.49)	286	0.43 (0.38–0.48)	115	40.21 (34.48–46.14)	5,461	8.24 (8.03–8.45)	1,313	24.04 (22.91–25.20)
Total	1,060,643	34,747	3.28 (3.24–3.31)	7,957	22.90 (22.46–23.35)	26,044	2.46 (2.43–2.49)	6,770	25.99 (25.46–26.53)	2,722	0.26 (0.25–0.27)	1171	43.02 (41.15–44.90)	63,513	5.99 (5.94–6.03)	15,898	25.03 (24.69–25.37)

1 *The proportion of HBsAg- and HBeAg-positive couples among couples only husband HBsAg-positive*.

2 *The proportion of HBsAg- and HBeAg-positive couples among couples only wife HBsAg-positive*.

3 *The proportion of HBsAg- and HBeAg-positive couples among couples both HBsAg-positive*.

4 *The proportion of HBsAg- and HBeAg-positive couples among HBsAg-positive couples*.

Meanwhile, 15,898 of 63,513 HBsAg-positive couples (25.03%, 95% CI, 24.69%−25.37%) were HBsAg- and HBeAg-positive couple, one or both of whom were positive for HBsAg and HBeAg, with a higher proportion of HBeAg in couples who were only wife HBsAg positive than in couples who were only husband HBsAg positive (25.99% vs. 22.90%).

### Ethnic Disparities in HBsAg/HBeAg Status in Reproductive-Age Couples

**Figure 1:**
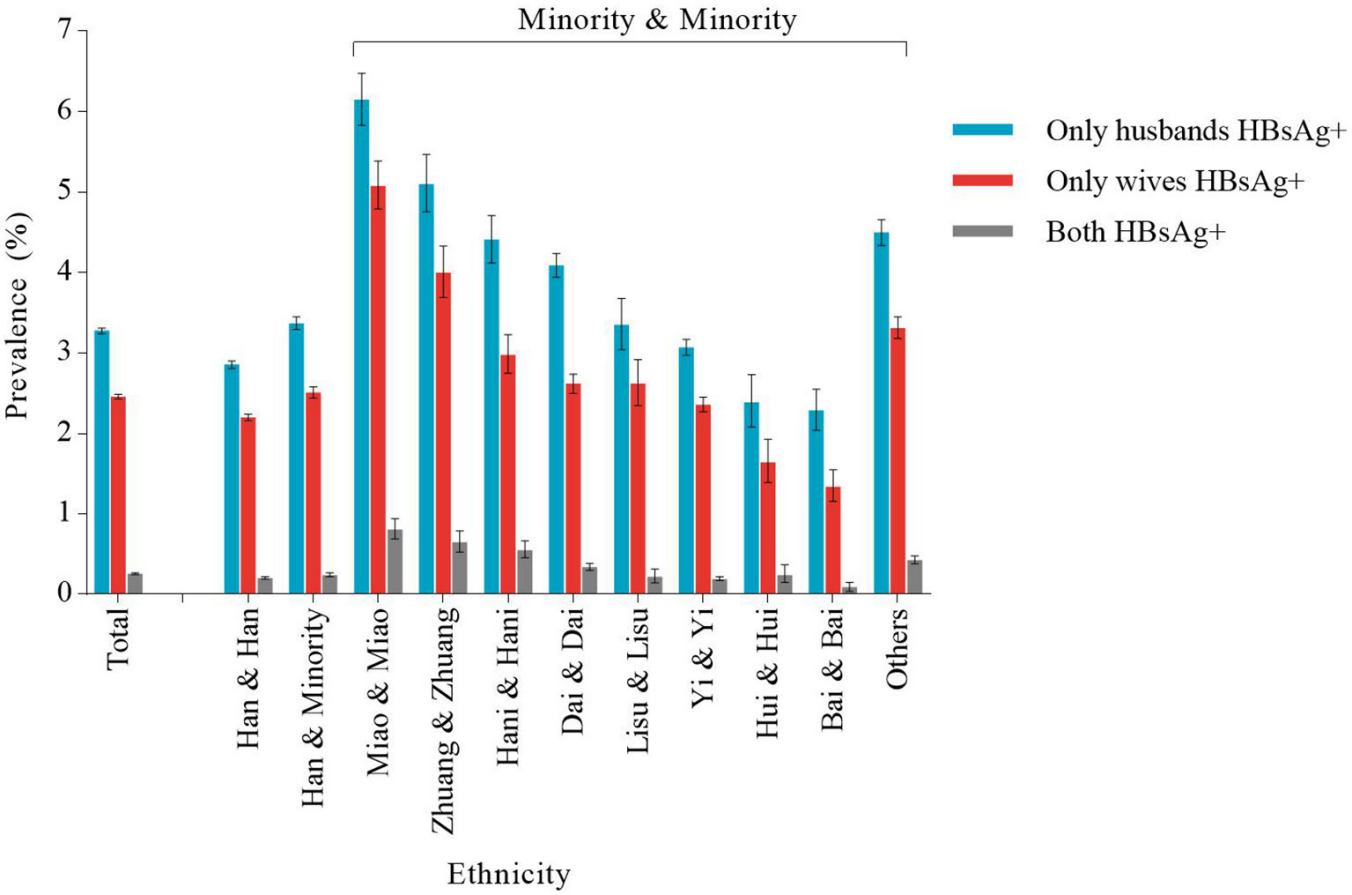
Ethnic disparities in hepatitis B surface antigen (HBsAg) status in reproductive-age couples.

The prevalence of HBsAg-positive couples of the Han and Minority ethnicity and Minority and Minority ethnicity (except the Hui and Hui ethnicity and Bai and Bai ethnicity) was higher than that of the Han and Han ethnicity, with the highest prevalence in the Miao and Miao ethnicity (12.04%, 95% CI, 11.61%−12.48%) and Zhuang and Zhuang ethnicity (9.76%, 95% CI, 9.29%−10.24%) (Table 2). In the prevalence of couples, only husband HBsAg positive was higher than that of couples only wife HBsAg positive across all ethnic groups (Figure 1). Meanwhile, the highest proportion of HBsAg- and HBeAg-positive couples among the HBsAg-positive couples was also observed in the Miao and Miao and the Zhuang and Zhuang ethnic groups (35.41% and 35.90%, respectively) (Table 2).

**Figure 2:**
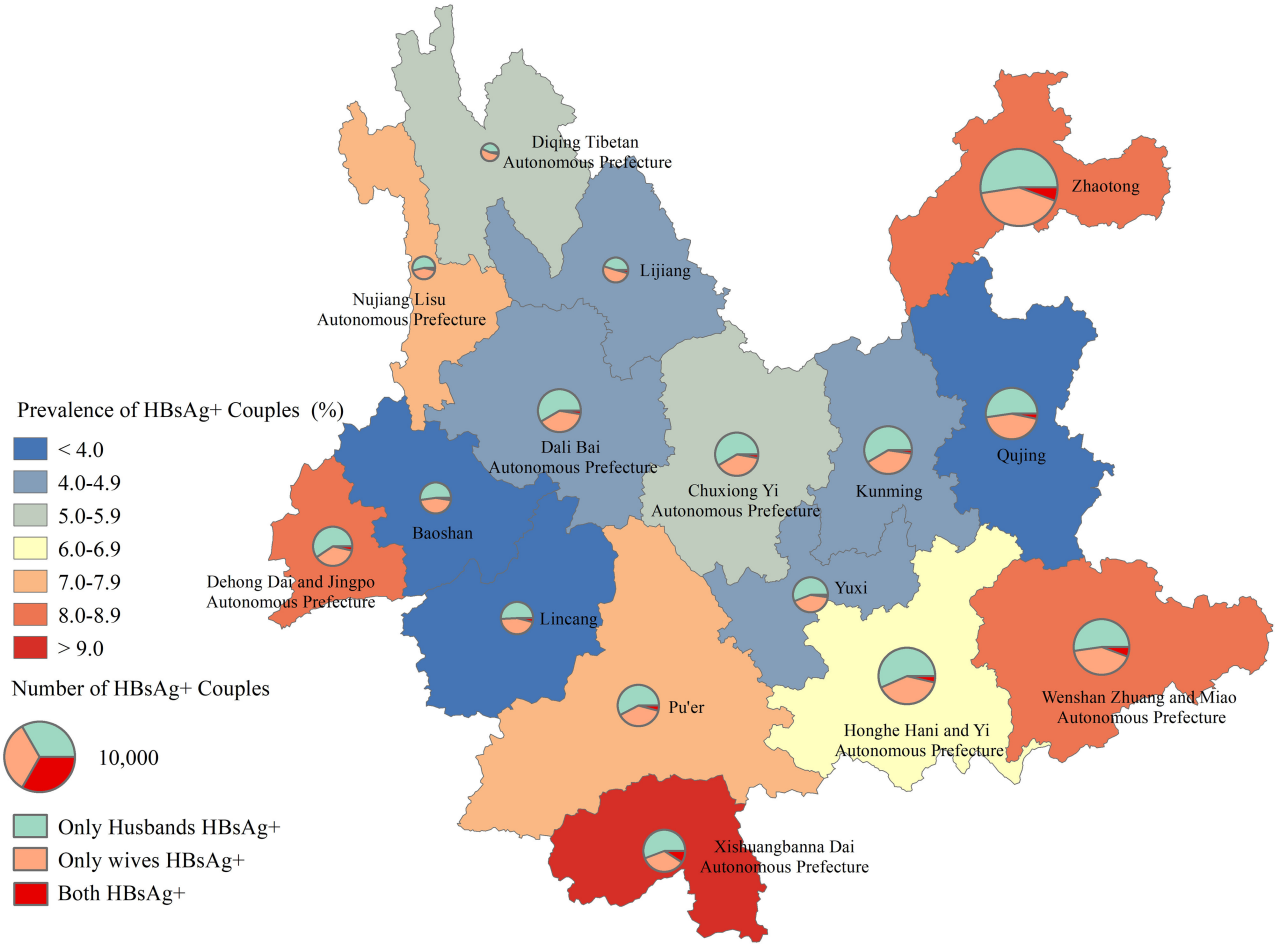
Geographic disparities in HBsAg status among reproductive-age couples in Yunnan Province, China.

According to the concentration of ethnic minorities in Yunnan, the prevalence of HBsAg-positive couples was also marked in maps to clearly show the ethnic and regional disparities in HBV infection among reproductive-age couples (Figure 2). At the municipal/ethnic autonomous prefecture level, the highest prevalence of HBsAg-positive couples was observed in Xishuangbanna Dai Autonomous Prefecture (12.70%), while the lowest was in Baoshan city (3.34%). In Xishuangbanna, more than 65% of the reproductive-age couples preparing for pregnancy were of the Dai and Dai ethnicity, Hani and Hani ethnicity, and other minorities and minorities. The prevalence of HBsAg-positive couples in these three ethnic groups was over 7% (Table 2). Although most of the reproductive-age couples preparing for pregnancy of the Zhuang and Zhuang ethnicity and Miao and Miao ethnicity gathered in Wenshan Zhuang and Miao Autonomous Prefecture, there were also a large number of Han ethnicity. The prepregnant couples of the Zhuang and Zhuang ethnicity and Miao and Miao ethnicity only accounted for ~30% of Wenshan Zhuang and Miao Autonomous Prefecture couples, while over half of the prepregnant couples were of Han and Han ethnicity and Han and Minority ethnicity.

### Association Between HBV Status of Spouses and HBsAg Status of Wives/Husbands

The HBsAg prevalence in wives increased with the positive status of HBsAg and HBeAg of their husbands, and the HBsAg prevalence in husbands also increased with the positive status of HBsAg and HBeAg of their wives (Table 3). The results from the multivariate logistic regression model showed that compared with the HBsAg prevalence of wives with HBsAg-negative husbands (2.55%), the HBsAg prevalence of wives with HBsAg-positive husbands (6.61%, aOR = 2.53, 95% CI, 2.41–2.65) and wives with HBsAg- and HBeAg-positive husbands (9.41%, aOR = 3.71, 95% CI 3.45–3.99) was significantly higher, after adjustment for ethnicity, age group, education, occupation, SES, and check-in year of the couples. Similarly, compared with the HBsAg prevalence of husbands with HBsAg-negative wives (3.37%), the HBsAg prevalence of husbands with HBsAg-positive wives (9.12%, aOR = 2.69, 95% CI 2.56–2.82) and husbands with HBsAg- and HBeAg- positive wives (10.43%, aOR = 3.10, 95% CI 2.88–3.35) was significantly higher.

**Table 3:** Association between HBV status of spouses and HBsAg status of wives/husbands.

**HBV status of spouses**	**Wives**			**Husbands**		
	**Positive (%)**	**cOR (95% CI)**	aOR^*^(95% CI)	**Positive (%)**	**cOR (95% CI)**	aOR^*^(95% CI)
HBsAg(–)	26044 (2.55)	1 (Reference)	1 (Reference)	34747 (3.37)	1 (Reference)	1 (Reference)
HBsAg(+) and HBeAg(**–**)	1895 (6.61)	2.71 (2.58–2.84)	2.53 (2.41–2.65)	1934 (9.12)	2.88 (2.74–3.02)	2.69 (2.56–2.82)
HBsAg(+) and HBeAg(+)	827 (9.41)	3.98 (3.70–4.28)	3.71 (3.45–3.99)	788 (10.43)	3.34 (3.10–3.60)	3.10 (2.88–3.35)

* *Adjusted by ethnicity, age group, education, occupation, socioeconomic status, and check-in year of the couples*.

### Association Between Ethnicity and HBsAg Status of Wives/Husbands

**Figure 3:**
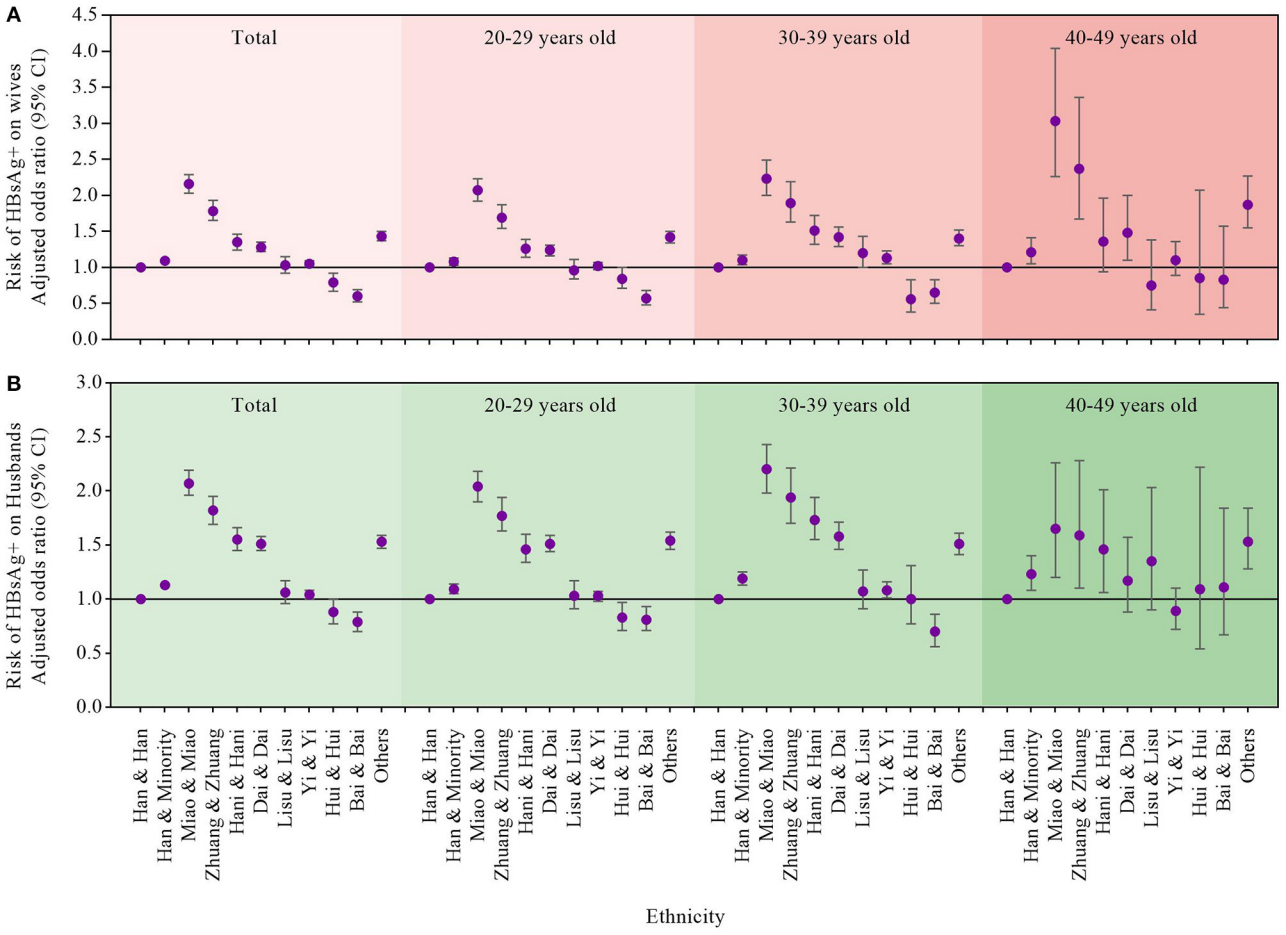
Multivariate analyses of the association **(A)** between ethnicity and HBsAg status of wives **(B)** between ethnicity and HBsAg status of Husbands. Han and Han served as the reference group. Multivariate analyses were adjusted by age group, education, occupation, socioeconomic status and check year of the couples, and HBV status of **(A)** husbands/**(B)** wives.

The results from the multivariate logistic regression model showed that the risk of HBV infection of wives in the Miao and Miao (aOR = 2.16, 95% CI, 2.03–2.29), Zhuang and Zhuang ethnic groups (aOR = 1.78, 95% CI, 1.65–1.93), and other minorities (aOR = 1.43, 95% CI, 1.37–1.50) was significantly higher than that in the Han and Han ethnicity, after adjustment for age group, education, occupation, SES, and check-in year of the couples, and HBV status of husbands (Figure 3A). Stratified analyses by the age group of the couples (20–29, 30–39, and 40–49 years old) were also performed, and the results were relatively stable and showed that the ethnic disparities decreased with the decreasing age group in the multivariate logistic mode after adjustment for education, occupation, SES, and check-in year of the couples, and HBV status of husbands. Compared with the majority of Han and Han ethnicity, the risk of HBV infection in the Miao and Miao ethnicity decreased from 3.03 (95% CI 2.26–4.04) times in the 40–49-year group to 2.07 (95% CI 1.92–2.23) times in the 20–29-year group.

Similarly, the risk of HBV infection of husbands in the Miao and Miao (aOR = 2.07, 95% CI, 1.96–2.19), Zhuang and Zhuang (aOR = 1.82, 95% CI 1.69–1.95), Hani and Hani (aOR = 1.55, 95% CI 1.45–1.66), other minorities (aOR = 1.53, 95% CI 1.47–1.59), and Dai and Dai (aOR = 1.51, 95% CI 1.45–1.58) ethnic groups was significantly higher than that in Han and Han ethnicity, after adjustment for age group, education, occupation, SES, check-in year of the couples, and HBV status of wives (Figure 3B). Stratified analyses by the age group of the couples (20–29, 30–39, and 40–49 years old) were also performed and showed that the ethnic disparities decreased in the 20–29-year group compared with the 30–39-year group in the multivariate logistic mode after adjustment for education, occupation, SES, check-in year of the couples, and HBV status of husbands.

## Discussion

We performed a population-based, cross-sectional study in rural Yunnan, China, to understand the ethnic disparities in HBV infection among 1 million reproductive-age couples to provide reliable data for equitably eliminating hepatitis B. To our knowledge, this is the first study to describe ethnic disparities in HBV prevalence among couples as a unit in China, and the findings will lead to identifying priority ethnic groups, developing evidence-based strategies targeting intrafamilial HBV transmission, increasing health equities within the hepatitis response in China, and contributing to the realization of WHO goals of eliminating hepatitis B by 2030.

In total, almost 6% of 1 million reproductive-age couples were HBsAg-positive couples, and 25% of the HBsAg-positive couples were also positive for HBeAg in rural Yunnan. The prevalence of HBsAg-positive couples in Yunnan in 2014–2019 was lower than that in rural China in 2010–2012, with nearly one in ten couples as a source of HBV infection (7). The reason was that Yunnan is a province in Southwest China, and the HBsAg prevalence was significantly lower in the central or western regions than in the eastern regions in China (14). Additionally, the proportion of HBsAg- and HBeAg-positive couples was consistent with a previous national study in which ~27.84% of HBsAg-positive couples tested positive for HBeAg (14). HBeAg positivity indicates a high level of infectiousness and has high sensitivity to predict infants with immunoprophylaxis failure (19). Because HBV can be transmitted by sexual transmission or by vertical transmission, HBsAg-positive couples preparing for pregnancy, especially HBeAg-positive couples, deserve more attention, particularly in rural areas where financial and medical resources are relatively limited (7).

For ethnic groups, the Miao and Miao ethnicity and Zhuang and Zhuang ethnicity have the highest prevalence of HBsAg positive couples (12.04% and 9.76%) and the highest proportion of HBsAg- and HBeAg-positive couples (35.41% and 35.90%) in rural Yunnan. The risk of HBV infection of either wives or husbands in the Miao and Miao ethnicity and Zhuang and Zhuang ethnicity was significantly higher than that in the Han and Han ethnicity. These results were consistent with previous observational studies (10, 12). There were several explanations. First, some ethnic customs, such as walking marriage in Miao and Zhuang ethnic groups, may lead to high-risk behaviors, increasing the risk of HBV horizontal transmission (6, 20). Second, the coverage of the Hepatitis B vaccine varied among ethnic groups and regions due to the economic, cultural and medical barriers (6). For instance, the coverage was low in Wenshan Zhuang and Miao Autonomous Prefecture because of the large number of poor people in the past (21). Third, the HBV incidence was higher in the Miao and Zhuang ethnic groups than in the other groups (21), resulting in more new HBV-infected people being positive for HBeAg. Meanwhile, our results showed that in the prevalence of couples, only HBsAg-positive husband was higher than that of couples with only HBsAg-positive wife across all ethnic groups. These results were consistent with previous studies that HBsAg prevalence was higher in men than that in women (14, 22), because HBV infections before 2 years of age lead to chronic infections of 77% in men and 50% in women (23, 24).

For intrafamily horizontal transmission, our results indicated that the HBsAg prevalence of either wives or husbands rose with positive statuses for HBsAg and HBeAg of the spouse, and these associations remained stable after adjustment for other possible confounders. Currently, a strategy focusing on screening pregnant women might miss HBV-infected husbands who may be a source of infection in household settings in China. These findings indicated that to prevent the HBV intrafamilial horizontal and vertical transmission, positive couples should be screened and treated as an important unit before pregnancy. Susceptible spouses should be vaccinated against hepatitis B to prevent intrafamilial horizontal transmission (7). Susceptible wives of HBV-infected husbands should be vaccinated to prevent mother-to-child transmission of HBV. A previous study proved that the couple-based immunization strategy, including additional HBV screening for pre-pregnant couples and a hepatitis B vaccine for high-risk wives, can improve the efficiency of preventing HBV mother-to-child transmission, and is highly cost-effective (25).

For stratified analyses by age group, our results were relatively stable and showed that the ethnic disparities in HBsAg prevalence of the couples decreased with decreasing age group in the multivariate logistic model. The reason was that the Chinese government began to recommend the hepatitis B vaccine for the routine immunization of infants in 1992 (4). Then, the national coverage with a third-dose vaccine for infants increased from 30% in 1992 to 99.58% in 2015, and the national coverage with a timely birth-dose vaccine increased from 22.2% in 1992 to 95.61% in 2015 (4, 6). National surveys showed that individuals aged 15–29 years old had an HBsAg positive rate of 9.8% in 1992, 8.4% in 2006, and 4.4% in 2014 in China (26). The dramatic increase in coverage with the hepatitis B vaccine and a significant decrease in HBV prevalence means that more ethnic minorities in remote areas are gradually gaining access to these health services, leading to gradually narrowing gaps in HBV infection between the different ethnic groups in the younger population. However, currently, there are still obvious ethnic disparities in HBV infection in China.

There are several limitations of this study. First, Yunnan, a multiethnic province in Southwest China, differs from other provinces in population structure, perhaps leading to the limited representativeness. Second, the data in this study did not include risk factors related to intrafamilial transmissions, such as HBV status before marriage and years of marriage, so it was difficult to draw a causal inference in this cross-sectional study. Third, Hepatitis B vaccination history was not included in the final analysis considering the large recall bias of the self-reported history, and the difficulty of accessing official records. Fourth, the data did not include HBV genotypes, DNA load, or other risk factors, such as blood transfusions and family history of HBV infection.

## Conclusion

In conclusion, the HBV prevalence in reproductive-age couples was intermediate in rural Yunnan, China. Overall, 6% of 1 million couples preparing for pregnancy were HBsAg-positive couples, and 25% of the HBsAg-positive couples were HBsAg- and HBeAg- positive, with the highest prevalence in the Miao and Miao ethnicity and Zhuang and Zhuang ethnicity. The HBV prevalence of either wives or husbands rose with positive statuses for HBsAg and HBeAg of the spouse. These findings indicated that there are still large ethnic disparities in HBV infection in China and large gaps in the WHO goals of eliminating hepatitis B. Positive couples should be screened and treated as an important unit before pregnancy to block HBV intrafamilial horizontal and vertical transmission, and ethnic minorities should be given priority to further equally eliminate hepatitis B as a public health threat by 2030.

## Data Availability Statement

The data that support the findings of this study are available from Yunnan Population and Family Planning Research Institute, but restrictions apply to the availability of these data, which were used under license for the current study, and so are not publicly available. Data are however available from the authors upon reasonable request and with permission of Yunnan Population and Family Planning Research Institute. Requests to access the datasets should be directed to Min Liu, liumin@bjmu.edu.cn.

## Ethics Statement

The study was approved by Institutional Review Board of Chinese Association of Maternal and Child Health Studies (Project identification code AMCHS-2014-6) and Biomedical Ethics Committee of Peking University (Project identification code IRB00001052-18100). The patients/participants provided their written informed consent to participate in this study.

## Author Contributions

ML and HY conceived and designed the manuscript. WJ did a literature search, analysis and interpretation, compiled tables and figures, and drafted the manuscript. YY, CK, and HY collected the data. JL and YW cleared the data and proofed the report. All authors participated in data analysis, interpretation, discussion, and writing of the manuscript.

## Funding

This study was supported by a grant from the National Natural Science Foundation of China (Nos. 71874003 and 71934002) to ML. The funders had no role in study design, data collection and analysis, decision to publish, or preparation of the paper.

## Author Disclaimer

The views expressed in the report are those of the authors and do not necessarily reflect the official policy or position of the Yunnan Population and Family Planning Research Institute.

## Conflict of Interest

The authors declare that the research was conducted in the absence of any commercial or financial relationships that could be construed as a potential conflict of interest.

## Publisher's Note

All claims expressed in this article are solely those of the authors and do not necessarily represent those of their affiliated organizations, or those of the publisher, the editors and the reviewers. Any product that may be evaluated in this article, or claim that may be made by its manufacturer, is not guaranteed or endorsed by the publisher.
